# Load Measurement in Linear Guides for Machine Tools

**DOI:** 10.3390/s19153411

**Published:** 2019-08-03

**Authors:** David Krampert, Sebastian Unsleber, Christoph Janssen, Leonhard Reindl

**Affiliations:** 1 Bosch Rexroth AG, 97421 Schweinfurt, Germany; 2 Institut für Mikrosystemtechnik, Albert-Ludwigs-Universität Freiburg, 79110 Freiburg, Germany

**Keywords:** piezoresistive, load measurement, linear guide

## Abstract

Process force determination in machine tools is an important topic for both optimization of manufacturing processes as well as predictive maintenance purposes. This work aims at providing a short overview of existing methods, motivating the integration of sensors into a linear guide, and then showing the results of an implemented new method for capturing the load on a rolling element linear guide by measuring the stresses resulting from the rolling element contact at the side of the runner block. The implementation of the approach is based on the piezoresistive diamond like carbon (DLC) coating DiaForce^®^ provided by the Fraunhofer Institute for Surface Engineering and Thin Films (IST), Braunschweig, Germany. Good agreement of measurements with analytical and finite element method simulation results promises a stable modeling and load estimation process. Nevertheless, non-idealities due to high temperature dependency and cross-sensitivity to non rolling element contact related stresses could be shown, but also an efficient approach to diminish these effects is included.

## 1. Introduction

Force measurement in machine tools can be viewed from two application perspectives: either for error compensation during the manufacturing process or for prediction of the failure of one or several components. Process forces lead to deformation of the machine structure and subsequently cause, if they are not accounted for, deviations from the ideal tool path. Ramesh et al. [1], for example, show the implications of cutting force on the error in the final product. Denkena et al. successfully use sensors in notches manufactured into the spindle slide in [2], in order to compensate tool deflection. In predictive maintenance applications, where the remaining lifetime of a component has to be determined, one major factor is the mechanical load on the component. This is usually estimated in the design process of the machine. But deviations either due to tolerances during the manufacturing of the machine, as well as ageing processes or external factors such as e.g., changes in the foundation below a heavy machine and therefore in the constraining forces, will lead to an over or underestimation of the remaining lifetime.

For error compensation, Rao et al. [3] distinguish three kinds of methods: process design, off-line compensation approaches and on-line compensation approaches. Equivalent concepts can be defined for the predictive maintenance aspect. The process design approach simply increases the safety margin during the design process, leading to non-ideal performance of the machine. Off-line compensation approaches try to predict the error from either analytical models [3] or purely empirical methods, by e.g., training neural networks [4]. On the positive side, off-line approaches only require a one time effort before deploying the system, having no need for additional hardware like e.g., sensors. As a drawback, the accuracy of off-line methods highly depends on the assumptions made during modeling and they can hardly react to environmental changes. Especially when the number of variables, such as process force, temperature, tool degradation etc. is high and interdependencies between the variables make the models very complex, a monitoring of the environment can improve results, as also mentioned in [5]. On-line approaches such as [6] reduce the number of necessary assumptions by directly monitoring the relevant variables. This promises a more reliable and stable error correction, at the cost of additional hardware and software required for precise data acquisition. The overhead effort could be reduced if the measurement system could be integrated into a standardized, commonly used component of the tooling machine. An additional challenge of on-line force monitoring approaches is integrating the sensors without significantly influencing the overall system performance.

Most methods use a transducer based on the elastic deformation of a material under load. This deformation is then transduced into a change of an electrical quantity. Commonly used and widely known methods are piezoelectric [7] or piezoresistive elements [8]. A comprehensive overview can be found in [9]. Aiming at diminishing the influence by the load determination system on the over all mechanical behavior is ruling out otherwise successful approaches like weakening the structure as proposed in [10] and adding intermediate components, such as a force ring [7]. Solutions applying sensors directly to or into the structure as in [11] enable on-line monitoring even under this restriction. A general methodology for integrating sensors into existing structures is described in [12], also further motivating the approach. Tooling machines exist for a variety of purposes, performing many different processes. The ideal case would thus be a standardized component, which is used in almost all machine tools and also is in the direct flux of force. Linear guide bearings qualify for both mentioned requirements (standardization according to ISO-12090-1 [13]). Additionally, the high loads and process forces linear bearings have to withstand make them subject to heavy wear, emphasized by the fact that they directly suffer from constraining forces e.g., resulting from miss-alignments. This makes them account for approx. 29% of feed axis failures in machine tools [14]. Therefore, a reliable, active predictive maintenance approach for linear guide bearings promises to greatly improve the machine’s cost and time efficiency.

Both predictive maintenance and process optimization can greatly benefit from load determination. For this, on-line load monitoring concepts can be expected to bear the most reliable data. Linear guide bearings are a suitable location for both goal applications. Finding a way to integrate such a sensor system into a linear guide bearing without interfering with the machine performance is the scope of this paper.

The work presented in this paper falls in the Industry 4.0 domain, in which many researchers [15] and companies of the machine building sector are currently active. For example a lubrication analysis unit exists for linear guides, or a unit detecting displacement of a rotatory bearing (See e.g., http://www.schaeffler-fairs.de/machinetool4.0/). Nevertheless, an integrated method for determining the load on a linear guide is not established yet. In this work we will present such a method, based on the stresses introduced by the rolling element contact, and deliver a model for the sensor signals. This model will be verified. Also, we will address and compensate disturbing signals, which are not related to those stresses.

### Structure

Since this paper is quite extensive, the structure will be explained in the following.

In Section 2.1 a quick introduction to linear guides is given. Then, an overview of existing concepts for related problems is presented, both for motivating this work further and narrowing the scope.

In Section 2.2, the main working principle of the load measurement system is explained by showing sensor location and physical behavior.

Having an analytical prediction of sensor behavior in the linear guide is crucial both for a deeper understanding and for actually evaluating the sensors signals. A model is provided in Section 2.2.1 and verified by comparison with finite element method (FEM).

The measurement setup for sensor analysis is shown in Section 2.2.2.

Evaluation of the sensor response showed non-idealities, which can be efficiently diminished. This is presented in Section 2.2.3, while the useful signal magnitude is shown in Section 2.2.4.

Section 2.2.5 shows that the measured signal is actually representing the rolling element contact related stress by comparison with simulation.

For practical applicability, one has to be able to retrieve the load from the sensor signal. In Section 2.2.6, the results of a statistical estimator are presented.

Finally, in Section 2.2.7, a remaining, yet to be compensated, non-linearity having hysteretic properties is shortly presented.

## 2. Results

### 2.1. Linear Guide Bearings

**Figure 1:**
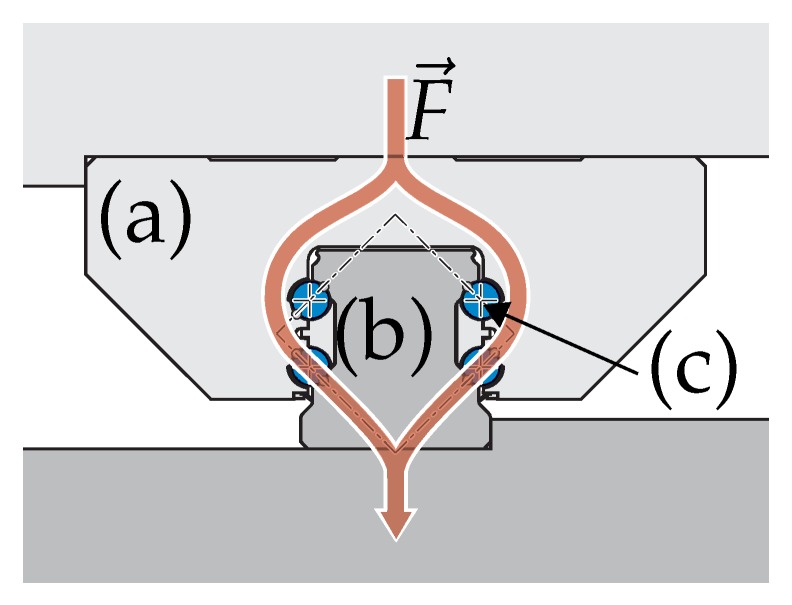
Sketch of a linear guide bearing with a runner block (**a**) on a rail guide (**b**) connected via rolling elements (**c**). With an exemplary force flux $\vec{F}$ [17].

Measuring the load on the linear guide bearing has been found to be a promising location inside the tooling machine. Linear guide bearings consist of a rail guide and a runner block, connected via rolling elements, which can be spherical, cylindrical or have similar, optimized surface curvatures [16]. A sketch is provided in Figure 1.

Adding additional parts as e.g., a force measuring platform was ruled out before for the scope of this paper, as it changes the mechanical properties and would also add to the standardized dimensions of the runner block. Thus, if one wants to deduce the load from deformation, the deformation of the linear guide system or its parts has to be measured directly. On this topic, or related problems in general rolling element bearings, mainly various patents exist. Only little scientific data, especially for linear guides, could be found, such that only a conceptual comparison is feasible at this point.

#### 2.1.1. Measuring the Deformation of the Runner Block

A load acting on the runner block leads to a deformation of the same, creating regions both with negative and positive strain. This strain can be measured by e.g., strain gauges, as already described and used in various patents [18] (without strain gauges) [19,20]. One challenge is the coupling of the strain gauges to the runner block, which will greatly influence the sensitivity of the sensor. Furthermore, in order to distinguish different multi modal load situations and achieve sufficient resolution, intelligent sensor placement and sensor fusion algorithms have to be developed.

#### 2.1.2. Measuring Deformation Related to the Rolling Element Contact Zone

Another region in the runner block is subject to large deformations. The force flux flows from the runner block, over the rolling elements to the rail guide. This leads to high and localized deformation of the contact zones between these three elements. Measuring this deformation is highly attractive for the predictive maintenance aspect, as load would be measured directly or very close to where damage due to fatigue occurs. Therefore uncertainties introduced by assumptions about the exact load distribution under multi modal external loads will be eliminated. Also tolerances in rolling element sizes, leading to higher loads for larger elements, can be directly accounted for.

##### Deformation of the Rail Guide

Winkelmann et al. [21] measure the deformation of the rail guide by applying, preferably piezoresistive, sensitive elements on it. It has to be mentioned that this deformation is not necessarily the direct deformation in the contact zone, but the sensor could also be located elsewhere, as the precise location is not specified in the patent. This approach fixates the sensor elements to a specific part of the railguide, or implies the usage of many sensor elements if one was to cover the whole railguide. This trades off easy contacting of the stationary sensor elements with flexibility regarding the process during which the load should be determined. Therefore the rail guide approach is favorable, if the machine performs processes with high loads occurring only at a specific proportions of the rail guide and non directly process related forces (like constraining forces) elsewhere are not of major interest. The decision to look for ways to integrate the sensor elements into a standard component was based on the implied flexibility, placing measurement at the runner block or the rolling elements in focus.

##### Deformation of the Rolling Elements

Aust and Mayer [22] measure the deformation of the rolling elements, or more precisely replace one of the elements by a sensor element, including peripherals for communicating the acquired data. Placing the sensor directly at the rolling element fulfills the requirement of being close to the point of actual load transfer and is fully flexible towards the applied process. As a drawback, it requires a significant amount of space to fit in the electronics, thus is aimed only at large scale bearings. This particular solution is also held in place by a cage, which is not present in all linear guide bearings. Conclusively, integrating sensor elements and peripherals inside the rolling elements can be seen as an efficient approach for a specific type of bearings, but can be problematic in the case of the linear guides, especially when considering applicability to different sizes.

##### Deformation at the Runner Block

No approach which aimed at measuring the strain or stress introduced by the rolling element contact on the side of the runner block has been found (other than the one which forms the basis of the following investigations, patent request [23]). The algorithmic challenges are similar to those of [21], where the sensors are applied to the rail guide.

For rotatory bearings, an implementation can be found (Piezoresistive Dünnschichtsensorik im direkten Wälzkontakt in Lagern von FAG. Website: https://www.adaptronik.fraunhofer.de/de/appl1/energ/smart/lagersensorik.html). In general, this proposes a similar setting to the linear guide problem at hand. Biehl et al. show a raw resistive signal of the sensory bearing in rotation and under load in [24]. This already demonstrates the principal feasibility of load measurement in rolling element bearings by strain sensors directly in or close to the raceway. Anyway, in this paper, transferability to a linear guide will be investigated. Further, the existence and compensation of cross-sensitivities, modeling of the sensor signal based on the input variables and a robust load estimation procedure will be addressed. These extensions are fundamental for the successful use of the sensor system.

##### Summary

To overcome the restrictions in the existing methods either measuring the deformation of the runner block as a whole or directly measuring the strain introduced by the rolling element contact seem very promising. Both topics are still open to basic research with no or little scientific data being available. In the end, the actual load transfer in the bearing happens at the contact of the rolling elements, which implies that one level of abstraction is needed less when modeling the latter approach. Therefore, this method was chosen for closer investigation.

### 2.2. Measuring Strain due to Contact Stresses

When measuring the stresses directly related to the contact zone, the applied sensor principle has to meet two fundamental requirements, high k-factor and high durability. The k-factor describes how much the sensor signal changes relative to the strain, i.e., change in size, caused by the force to be measured, as in Equation (1). When the sensor is put directly into the force flux, it should not require significant strain to produce measurable signal changes, otherwise the linear guide as a whole will yield beyond its specifications. On the other hand, stresses close to the contact center can reach high values, requiring a very rugged sensor itself and also reliable coupling to the steel substrate, otherwise e.g., delamination can occur. Taking this into consideration, the DiaForce^®^ coating by the Fraunhofer Institute for Surface Technology was chosen. A similar sensor was already successfully used by [25]. There it was used on a pin in a double nut arrangement to monitor preload. The sensor was not used to detect the current loading, or close to the rolling element contact, as it is in this work. The sensor is based on an amorphous carbon layer with high sp3/sp2 hybridization ratio, commonly referred to as diamond like carbon (DLC), which is known to exhibit significant piezoresistive behavior. The exact k-factor depends on the coating parameters and can reach values up to 1200 [26,27]. In order to calculate the mechanical stress from the electrical resistance, calibration data of the elements towards regarding the piezoresistive effect and the temperature dependence is required. The origin of the piezoresistive effect is not fully understood, but can be approximated to result in a linear dependency of the resistance *R* on the strain ε. Further the dependency of the resistance on the temperature *T* follows Arrhenius behavior. Both dependencies are shown in [28], yielding the following parametric equations for the calibration process:

(1) $$\begin{array}{cc} R(\varepsilon ) & =R_{0}\left(1+k\varepsilon \right)\\ R(T) & =R_{0}e^{B(\frac{1}{T}-\frac{1}{T_{0}})}, \end{array}$$

where $R_{0}$ is the reference resistance in the absence of (external) strain at reference temperature $T_{0}$, *k* is the gauge factor and *B* is the coefficient describing the temperature dependency. Ref. [28] also states the independence of the gauge factor on the temperature, at least in the range of 23 ${}^{\circ }$C < *T* < 60 ${}^{\circ }$C. DLC coatings have been already studied for use in bearings, e.g., in [29], and a thorough overview of their tribological properties is found in [30].

**Figure 2:**
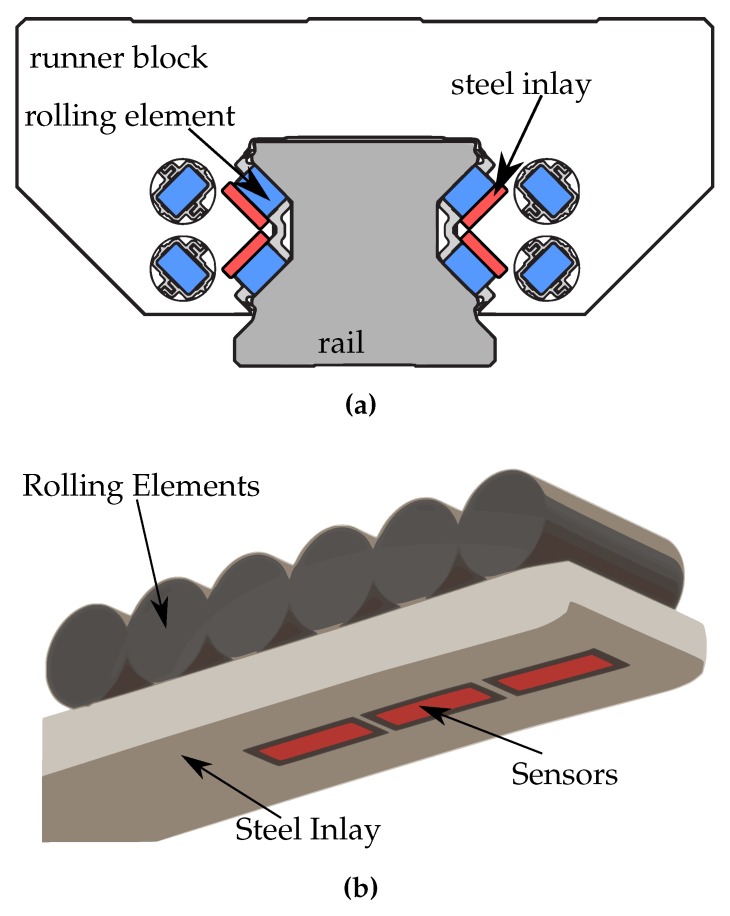
(**a**) Position of steel inlays (red) below the rolling elements (blue) inside the roller runner block, depiction from [17] (modified). (**b**) Sketch of the sensor position on the steel inlay (red).

The roller runner blocks manufactured by Bosch Rexroth exhibit a specialty, the steel inlay. This is a part of hardened bearing steel used for the actual raceway, glued onto the runner block body, as illustrated in Figure 2a. It was chosen to apply the coating to the backside, i.e., the side facing the runner block, as illustrated in Figure 2b.

On the backside, the sensors were still close to the contact zones, with the previously described benefits for load estimation, but are subject to milder stress levels (peak von-Mises equivalent stresses approx. only half of those in the direct contact according to FEM simulations), aiming for higher durability and longevity of the sensor system.

#### 2.2.1. Analytical Derivation of Stresses

The mechanical stresses caused by loading the runner block are dependent on numerous factors, e.g., the exact geometry of the runner blocks. Therefore an analytical derivation of the full model would be too involved for practical applications, making numerical FEMs more attractive. Anyway, an analytical solution can be derived for a highly simplified case, which is still useful for a general analysis of the problem, showing the dependencies of the mechanical stress, i.e., also the sensor signals, on the applied force and relative position of the rolling elements. The sensor data was still evaluated against FEM simulation data later on.

**Figure 3:**
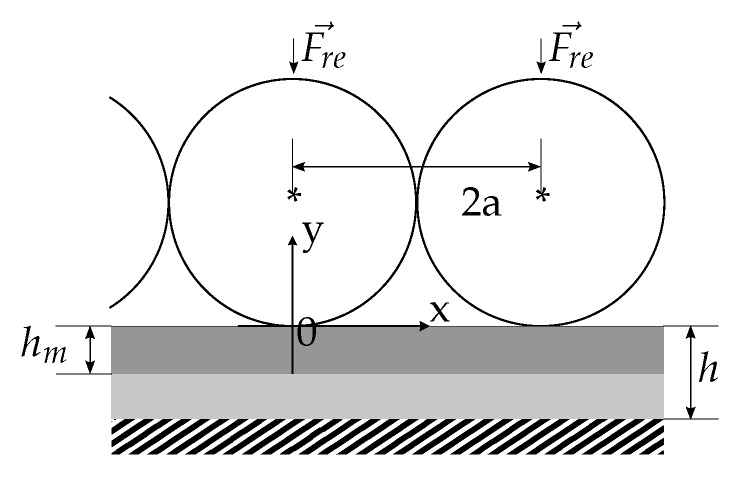
Sketch of the simplified two dimensional model of the rolling element problem for analytical evaluation.

The overall procedure described in the following is largely based on the work for a single body/layer in [31], extending it by the notion of using joint boundary conditions to model multiple connected bodies briefly mentioned in [32]. This way, the steel inlay can be modeled to rest upon an elastic body (i.e., the runner block), which in turn, for simplicity, rests on a rigid foundation. In reality, the linear guide system will deform when load is applied. This contradicts the rigid foundation assumption. Bringing the sensor close to the rolling elements should diminish such effects. The validity of this assumptions will be checked by comparing the analytical results to FEM data derived from a more realistic model. For the simplified case a single, infinitely long steel inlay was assumed, on which a periodic load, i.e., by infinitely many, equally spaced cylindrical rolling elements, is applied. Further, the problem was reduced to a two dimensional one by assuming the rolling elements and the steel inlay to be infinitely wide and the stresses to be constant along this axis. A section of the problem is sketched in Figure 3 with the corresponding coordinate system. On the *x*-axis the elements will occur periodically until infinity.

There are 8 boundary conditions to be defined. Traction boundary conditions were chosen such that shear stresses $\sigma_{xy}$ are 0 at all body interfaces. Displacement $u_{y}$ in y direction was forced to 0 at y=−h by the rigid foundation. Displacement as well as stress $\sigma_{y}$ in the y-direction was modeled to be ideally transferred between the two bodies, i.e., were set as equal. The stresses at the contact with the rolling elements was given according to a model chosen for the contact zone, leading to pressure profile p(x). For simplification, the pressure profile applied by the individual rolling elements was modeled to be uniformly distributed over the contact width. This represents a periodic rectangular pulse, the width of which is derived from the classical contact theory [16] as $b=2\sqrt{\frac{8F_{re}a\left(1-\nu^{2}\right)}{\pi El}}$, where $F_{re}$ is the loading force per rolling element, *a* is the radius of the rolling elements, ν is Poisson’s Ratio, *E* is Young’s Modulus and *l* is the real length of the rolling elements. This leads to more convenient expressions in the Fourier domain than the original half-circle pressure distribution, especially simplifying the back-transformation step. The pressure p(x) is then defined as

(2) $$\begin{array}{cc} p_{b}\left(x\right) & =\left[\eta \left(x+\frac{b}{2}\right)-\eta \left(x-\frac{b}{2}\right)\right]\frac{F_{re}}{bl}=\left[\eta \left(x+\frac{b}{2}\right)-\eta \left(x-\frac{b}{2}\right)\right]p_{0} \end{array}$$

(3) $$\begin{array}{cc} p(x) & =\sum_{k=-\infty }^{\infty }p_{b}\left(x-2ka\right), \end{array}$$

where η is the Heaviside step function.

The problem is then solved by Fourier transformation of the pressure profile and the governing Navier-Lamé equations and solving for the displacement *u* leading, together with the boundary conditions, to an equation system, linear in the coefficients to be determined (again, see [31] for the derivation for a single body):

(4) $$\begin{array}{cc} u_{x,1}\left(x,y\right) & =\left[A_{11}+\left(A_{11}-iA_{21}\right)\frac{sy}{\kappa }\right]e^{sy}+\left[B_{11}-\left(B_{11}+iB21\right)\frac{sy}{\kappa }\right]e^{-sy}\\ u_{y,1}\left(x,y\right) & =\left[A_{21}-\left(A_{21}+iA_{11}\right)\frac{sy}{\kappa }\right]e^{sy}+\left[B_{21}+\left(B_{21}-iB11\right)\frac{sy}{\kappa }\right]e^{-sy}\\ u_{x,2}\left(x,y\right) & =\left[A_{31}+\left(A_{31}-iA_{41}\right)\frac{sy}{\kappa }\right]e^{sy}+\left[B_{31}-\left(B_{31}+iB41\right)\frac{sy}{\kappa }\right]e^{-sy}\\ u_{y,2}\left(x,y\right) & =\left[A_{41}-\left(A_{41}+iA_{31}\right)\frac{sy}{\kappa }\right]e^{sy}+\left[B_{41}+\left(B_{41}-iB31\right)\frac{sy}{\kappa }\right]e^{-sy} \end{array}$$

relating to strains ε and stresses σ via

(5) $$\begin{array}{c} \varepsilon_{x}=\frac{\partial u_{x}}{\partial x},\varepsilon_{y}=\frac{\partial u_{y}}{\partial y},\varepsilon_{xy}=\frac{1}{2}\left(\frac{\partial u_{x}}{\partial y}+\frac{\partial u_{y}}{\partial x}\right)\\ o_{x}=\lambda \left(\varepsilon_{x}+\varepsilon_{y}\right)+2\mu \varepsilon_{x},\lambda \left(\varepsilon_{y}+\varepsilon_{x}\right)+2\mu \varepsilon_{y},\sigma_{xy}=2\mu \varepsilon_{xy} \end{array}$$

**Figure 4:**
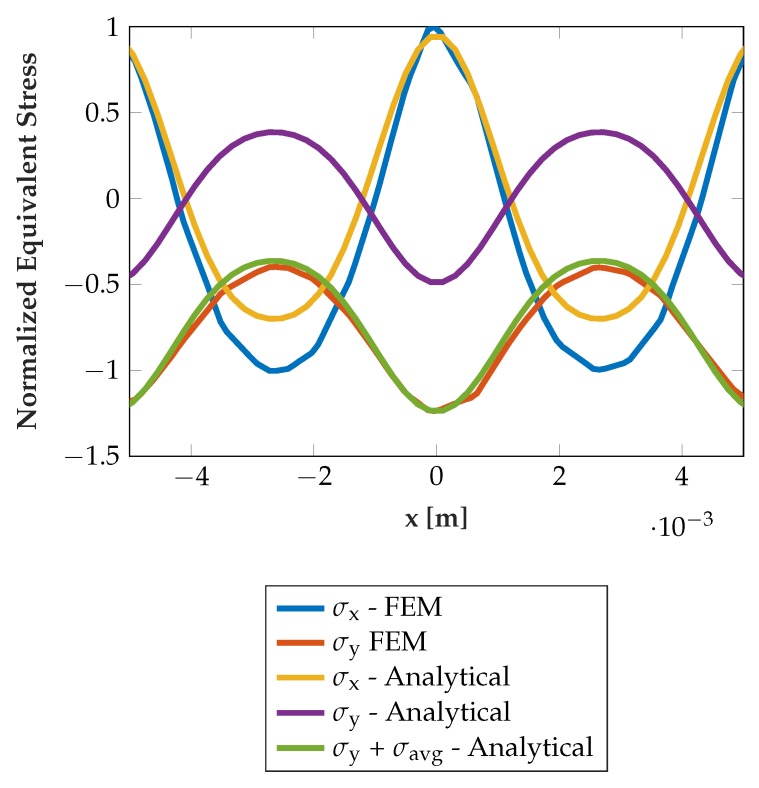
Comparison of stresses, determined analytically and by finite element method (FEM) simulation.

The solution is then transferred back into the spatial domain. Following all those steps, one obtains a series representation of the stresses in the spatial domain as (here just the normal stresses):

(6) $$\sigma_{x}\left(x,y\right)=\sum_{k=-\infty }^{\infty }\sigma_{x,k}\left(x,y\right),\sigma_{y}\left(x,y\right)=\sum_{k=-\infty }^{\infty }\sigma_{y,k}\left(x,y\right),$$

with

(7) $$\begin{array}{c} \sigma_{x,k}\left(x,-h_{m}\right)=\frac{1}{\tau }\sin\left(\frac{\pi bk}{2a}\right)p_{0}\cos\left(\frac{\pi kx}{a}\right)\\ \;(\left(\lambda +\mu \right)\left(2\nu -1\right)\left(\pi ka\left(\rho +\phi \right)+a^{2}\psi \right)+\\ \;\pi k\mu h_{m}(4\pi k\alpha \beta^{2}\left(\left(h_{m}-h\right)\left(\beta^{2}+1\right)\right)+\\ \;2a\beta^{2}\left(\beta^{2}+\alpha \left(\beta^{2}-\alpha -1\right)\right)))\\ \sigma_{y,k}\left(x,-h_{m}\right)=\frac{1}{\tau }\sin\left(\frac{\pi bk}{2a}\right)p_{0}\cos\left(\frac{\pi kx}{a}\right)\\ \;(\left(\lambda +\mu \right)\left(2\nu -1\right)\left(\pi ka\left(\rho +\phi \right)+a^{2}\psi \right)+\\ \;\pi k\mu h_{m}(4\pi k\alpha \beta^{2}\left(\left(-h_{m}+h\right)\left(\beta^{2}+1\right)\right)+\\ \;2a\beta^{2}\left(-\beta^{2}+\alpha \left(-\beta^{2}+\alpha +1\right)\right))), \end{array}$$

where

(8) $$\begin{array}{cc} \alpha & =e^{\frac{2\pi hk}{a}}\\ \beta & =e^{\frac{\pi h_{m}k}{a}}\\ \phi & =h(-4\alpha \beta^{4}+4\alpha \beta^{2})\\ \psi & =\beta^{6}-\beta^{4}-\alpha^{2}\beta^{2}+\alpha \\ \rho & =\left(\beta^{6}+2\alpha \beta^{4}-\beta^{4}+\alpha^{2}\beta^{2}-2\alpha \beta^{2}-\alpha^{2}\right)h_{m}\\ \tau & =\mu \pi \beta k(2\pi^{2}k^{2}\left(h_{m}\left(h_{m}\left(\beta^{4}+2\alpha \beta^{2}+\alpha^{2}\right)-4h\alpha \beta^{2}\right)\right)+\\ & 2\pi ka(h_{m}\left(\alpha \left(\beta^{4}-\alpha -1\right)+\beta^{4}\right)+h\alpha \left(-\beta^{4}+1\right))+\\ & 2\alpha (\beta^{2}\left(\beta^{2}\left(\alpha +1\right)-\alpha^{2}-2\alpha -1\right)+\alpha^{2}+\alpha )), \end{array}$$

where λ and μ are Lamé’s constants. The formulas in Equation (7) only describe the stresses at $y=-h_{m}$, i.e., at the location of the sensors. This is for the sake of compactness of formulas at this point, but of course stresses at different depths can also be calculated with the same method. It can be shown that in our case of having a “thick” layer/steel inlay, i.e., of height $h_{m}$ in the order of magnitude of the periodicity *a* of the pressure profile, the higher order parts (|k|>2) of the signal vanish quickly and can thus be neglected for $y\to -h_{m}$. The sensor signal at a specific relative position of the rolling elements can then be derived by integrating over the sensor length in *x*-direction at $y=-h_{m}$, i.e., determining the equivalent mechanical stress. This stress manifests itself in the measurement resistance via the piezoresistive effect. The results are largely compliant to the FEM simulation results, as it can be seen in Figure 4. Only an offset on the stresses in y-direction exists in FEM data, $\sigma_{y}$, which is not present in the analytical solution of the simplified problem. The offset originates from the finite dimensions of the steel inlay in the real/FEM problem. Without torque being applied, this average stress $\sigma_{avg}$ is the load per raceway divided by the steel inlay area. Respecting this, the analytical results are very close to the FEM results. The term $\sin\left(\frac{\pi bk}{2a}\right)p_{0}$ predicts, consistently with the FEM results, near linear scaling of the stresses with the applied force for b≪a. Due to the dominance of the |k|=1 portions, the stresses also consistently follow a near sinusoidal wave in *x*-direction with a period of 2a, i.e., the spacing and diameter of the rolling elements. The model, in extended fashion, can also be used to analyze the influence of material properties and coupling of the sensor layer and any additional layer, as e.g., a thin glue layer below the steel inlay, by again applying joint boundary conditions.

The expected sensor signals can then be calculated from the strain-resistance relation in Equation (1) (easily transferable to a stress-resistance relation assuming isotropy of material properties) and corresponding calibration data for *k* and $R_{0}$. Formulating stress dependency was chosen as in the calibration setting, obtaining stress was more straight-forward than for strain. So far it has been shown that results obtained via FEM and the simplified analytical description are largely compliant. This implies that the assumption of the foundation being a rigid body is good enough for the model to deliver useful results in this setting. In the following, measurement data will be compared to FEM data. Compliance between FEM and measured data will also imply validity of the analytical derivation.

#### 2.2.2. Measurement Setup

**Figure 5:**
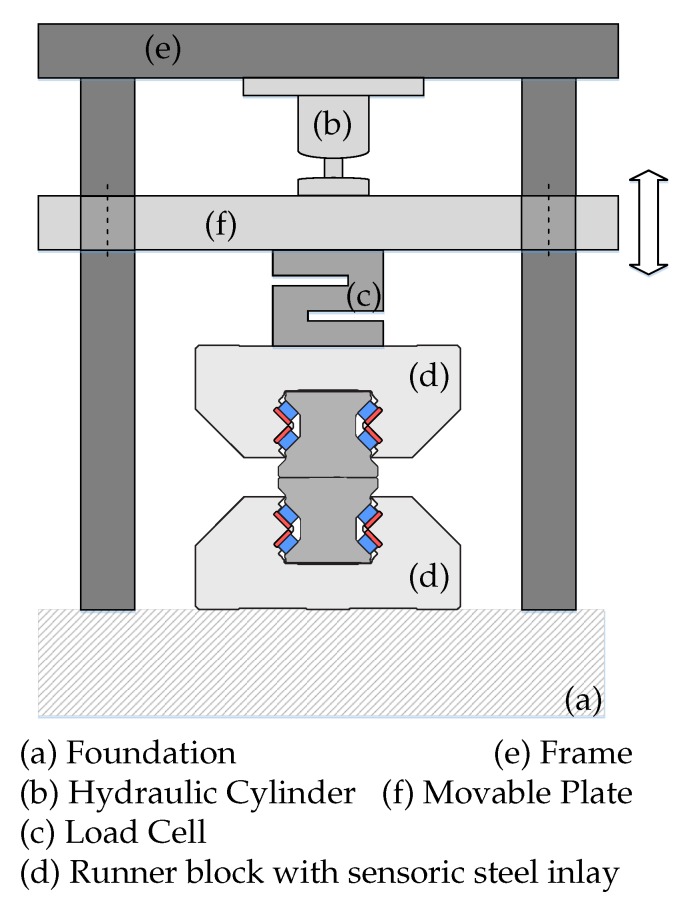
Sketch of the mechanical measurement setup. Runner block graphic from [17].

To evaluate the sensor performance and verify compliance with FEM models and analytical calculations, the runner blocks were loaded with a perpendicular, uniaxial load varying from 0 kN to 100 kN. The load was recorded using a proprietary strain gauge based load cell This cell was regularly calibrated and verified to provide minimum ±1 kN accuracy over the whole measurement range. The mechanical setup is sketched in Figure 5.

Since the sensor signal is dependent on the relative position of the rollers to the individual sensors, the runner block was moved 1 mm between load cycles, causing a 0.5 mm displacements of the rollers. A distance of 12 mm was covered in 13 repetitions. In order to generate more data per test run and facilitate the mechanical setup, two runner blocks on two rail guides, screwed together on there bases, were loaded simultaneously. This leads to a total of four raceways being in the force flux.

**Figure 6:**
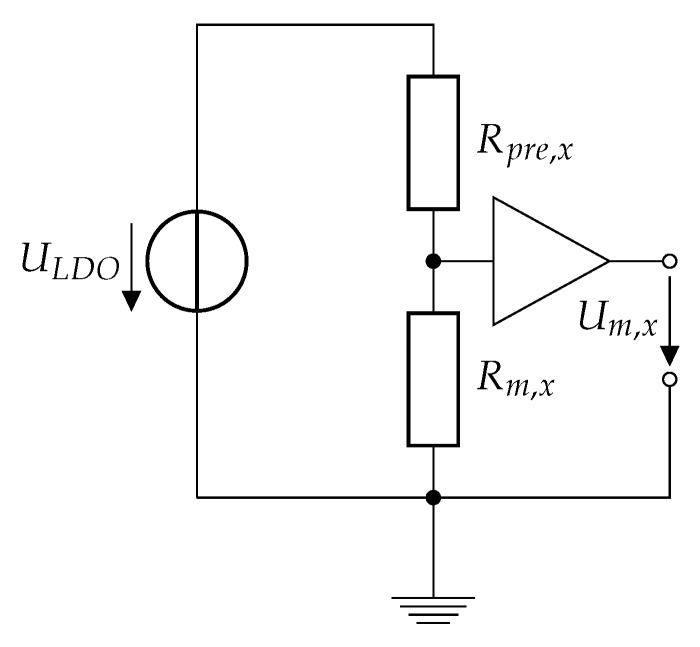
Electrical circuit used for sensor resistance evaluation.

For evaluating the sensor resistance of sensor element *x*, a shunt resistor $R_{pre,x}$ (temperature coefficient of resistance 50 $\frac{ppm}{K}$) was used to build a voltage divider. The circuit was fed by a voltage of $U_{LDO}=5$ V, supplied by a constant voltage low dropout regulator (LDO). The voltage $U_{m,x}$ over the resistance $R_{m,x}$ was then fed to a buffer and captured with a sampling frequency of 1 kHz. The whole circuit is depicted in Figure 6. Each sensor element has a dedicated shunt resistor and buffer element, but all were fed by the same voltage source. Since in the scope of this paper only near-stationary cases are considered (slow change rate of the applied force when standing still, $\frac{dF}{dt}\;<\;$5 kN/s, and slow velocity *v* < 50 $\frac{mm}{s}$ when in motion), only considering the real part of the signal, i.e., ignoring e.g., parasitic capacitances is feasible.

In order to be able to calculate an equivalent mechanical stress from the electrical resistance of the sensors, the sensor elements were provided together with calibration data. This is the sensor response to a direct perpendicular load, homogeneously applied to the sensor area. The result is two parameters, offset and slope, per sensor for a linear fit. Temperature dependency can be fitted to an exponential curve, using another two calibration parameters per sensor (see Equation (1)).

#### 2.2.3. Cross Sensitivity

As it was expected from the literature [24], the sensors show high temperature dependency. Further, due to the sensors’ amorphous nature on a micro scale, the macroscopically observed piezoresistive effect is largely isotropic, as also shown in [33]. Therefore, the sensor is not only sensitive to the directly load related strain introduced by the rolling elements, but also to the deformation of the runner block, or more precisely the steel inlay. Runner block deformation is dependent on additional external factors, e.g., how the moving machine part is mounted onto it. The decision in favor of this measurement method was based on presumable independence of such factors, which would take another layer of abstraction to compensate. A way to eliminate those stresses has to be found. Finally, when the rolling elements pass over the sensors, the obtained signal is highly dependent on the relative position of the rollers to the individual sensors (see Equation (7)). In order to measure the actual load, the roller location has to be estimated from the sensor signals as well.

**Figure 7:**
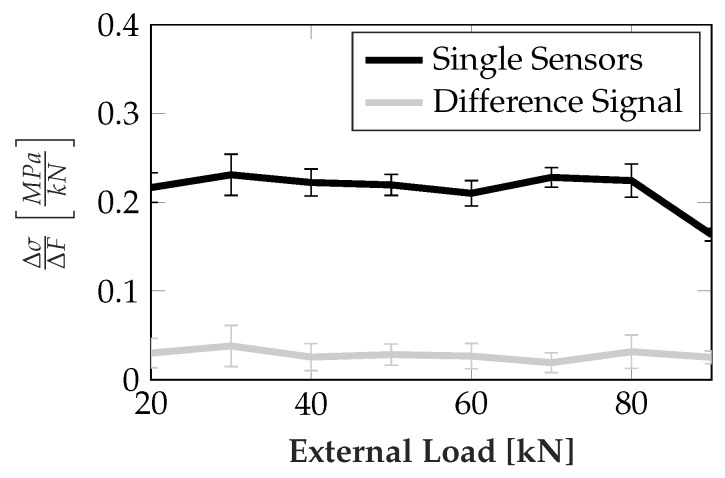
Mean change of sensor signals magnitude ± one standard deviation on unloaded raceways for individual sensors and the difference signal between neighboring sensors over an external load, after preload lifting.

The roller location sensitivity, if one is able to determine this location reliably, can be exploited to compensate most parts of both temperature dependency and cross-sensitivity, if the difference between two neighboring sensors is used instead of a single one. For this, the sensors have to be small enough for the roller position to have enough impact, i.e., should be in the approximate range of the roller size. The pairs also have to be very close. If this is given, any temperature will have nearly the same impact on the neighboring sensors, as will any stress introduced by global deformation of the runner block. In the case of the prototype the sensor size was 0.5 mm × 2 mm, where the 2 mm span was in direction of the roller movement, roller diameter was 5 mm. Compensation can then be achieved by numerically calculating the difference of the sensor pair signals and using this difference signal for further calculations. Alternatively, a sensor pair can already be electrically connected to form a bridge arrangement. For more flexibility the numerical difference was chosen in the prototype arrangement. In both cases, the effect is comparable to what is exploited in usual full-bridge strain gauge arrangements. Since the sensors are not trimmed for this, and trimming under load is very unlikely to be feasible, the sensors differ in their parameters. Thus full compensation with this method was not achieved. Nevertheless, the influence is highly reduced: for confirmation, the reduction of these parasitic stresses has to measured, which is hard to achieve directly, as it is superimposed with the actual rolling element related signal on the loaded raceways. When loading two raceways of the runner block with a perpendicular force on the same, the remaining two are unloaded. The runner blocks were preloaded for better mechanical characteristics, which means the raceways, which were not in the force flux, were unloaded after a certain amount of force was applied, which is called the point of preload liftoff from here on. For higher forces, no stress originating from the rollers can be measured by the sensors, making the unloaded raceways usable for assessing the cross-sensitivity compensation performance. The results are shown in Figure 7, depicting the change rate of the equivalent stress over an external load, at the point of maximum sensitivity regarding the rolling element position.

**Figure 8:**
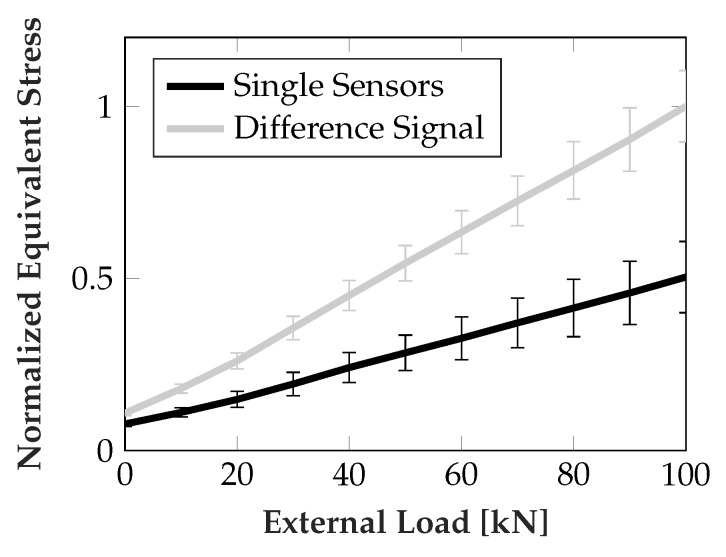
Comparison of sensor signal magnitudes for single sensors and difference signal.

The resistance of the sensor elements was converted to equivalent stress using a linear calibration curve obtained by direct surface perpendicular loading. Then the mean and standard deviation was calculated over all sensors on both runner blocks, each having four raceways with three sensor areas. No sensitivity towards the non rolling element related stress, i.e., the ideal case, would imply a change rate of 0 $\frac{MPa}{kN}$. From Figure 7 good compensation performance of the difference signal approach can easily be seen. The mean change rate over all evaluated load points was reduced from approx. 0.214 $\frac{MPa}{kN}$ to approx. 0.028 $\frac{MPa}{kN}$, equaling a reduction by approx. 86.9%. Additionally, an increased signal amplitude of the difference signal, as shown in Figure 8, further increases the ratio of useful signal to unwanted cross sensitivity.

**Figure 9:**
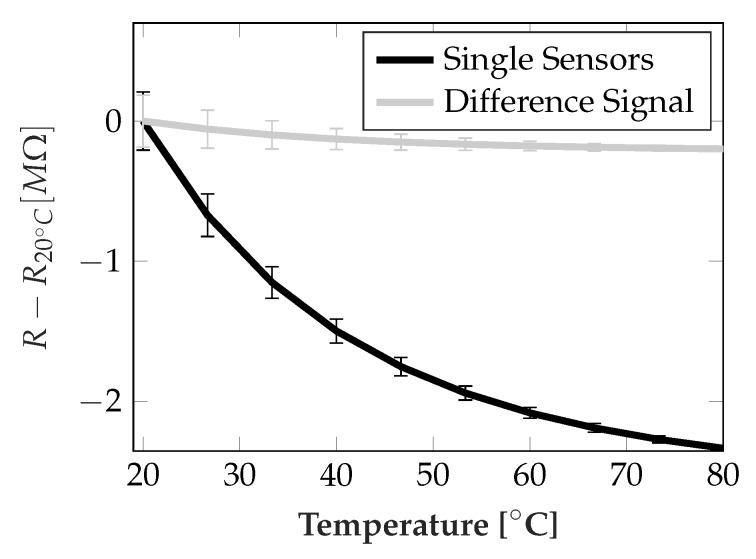
Mean reduction in temperature sensitivity by using difference signals, ± one standard deviation. From calibration data.

Figure 9 shows the performance of the temperature compensation achieved by using the difference signals.

The numbers are obtained directly from the calibration data for the exponential temperature dependency. The enormous predominance of the temperature dependent signal compared with load dependent signal made compensation by temperature measurement nearly infeasible even for small errors. A linearized mean temperature dependency from 20 °C to 80 °C of approx. $-3.31\;\frac{k\Omega }{{}^{\circ }C}$ using the difference signal compared to approx. $-38.9\;\frac{k\Omega }{{}^{\circ }C}$ using single sensors could be achieved. This reduction of temperature sensitivity by approx. 92.5% promises highly improved correction results.

#### 2.2.4. Signal Swing of the Difference Signal

It was shown that using the difference signal of a sensor pair leads to significant reduction of parasitic influences. Figure 8 shows the signal swing of individual sensors and difference signals. The mean ± one standard deviation of data obtained from the sensors on both runner blocks is depicted. Each runner block has two raceways in the force flux with three sensor areas each.

It can be seen that the achievable signal magnitude is nearly doubled, as the sensor signals change in positive and negative direction. This is a direct result of the isotropy of the piezoresistive effect and the three dimensional load case presented by the roller contact. The contact introduces both negative and positives stresses in different dimensions, which vary in magnitude periodically with the frequency of the rollers passing over the sensor. Since the sensor length is close to half of the roller diameter, this leads to improved signal swing when taking the difference of two neighboring sensors. The drawback is an increase in noise with a factor of $\sqrt{2}$ in its standard deviation, assuming additive, independent, normally distributed, zero mean noise of equal magnitude (standard deviation) at all the sensors, due to the subtraction of the two noisy signals.

#### 2.2.5. Comparison with Simulation

In order to prove the suggested measurement principle can be applied successfully, one has to show that the obtained signal is actually originating from the rolling element contact. The expected stresses were already described analytically, and compliance with FEM results was shown. If the experimental results agree with FEM results, and thus also the analytical model, the measurement method is successfully applied. In the simulation, the strain was obtained for a steel inlay without the coating, assuming neglectable influence on the overall mechanic response. During calibration unidirectional strain, perpendicular to the surface normal, is assumed. The exact relationship between simulated and measured signal might depend on e.g., deviations in material properties. Thus the following is meant to show agreement in shape, i.e., up to a factor of proportionality.

**Figure 10:**
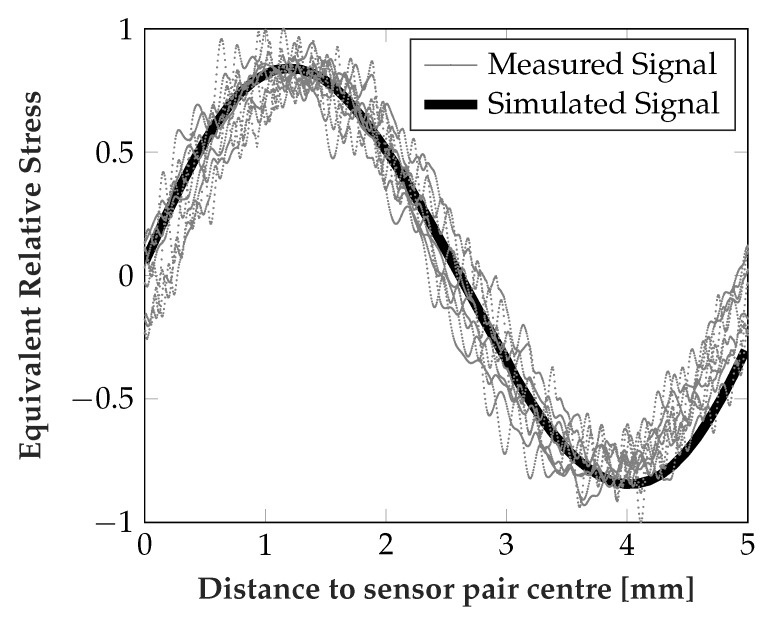
Simulated and measured difference signal at 50 kN with moving runner block for 100 mm.

The signal when the runner block is in motion is depicted in Figure 10, at an external load of approx. 50 kN. Here the signal was recorded for approx. 100 mm, leading to approx. 10 repetitions. The superposition after conversion to mechanical stress via calibration data and normalization is depicted. The measured difference signal actually showed an offset from the 0 MPa mean, probably indicating a small error in calibration data. This can be easily compensated also in a real application, as it was done for Figure 10.

**Figure 11:**
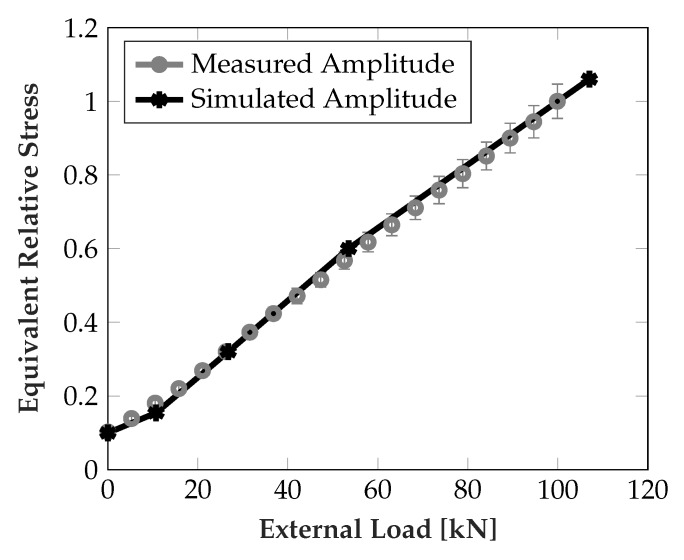
Simulated and measured difference signal amplitude at different loads, depicting one standard deviation for measurement data.

Figure 11 shows the simulated and measured signal swing of the difference signal over different external loads (simulated: 0, 0.1C, 0.25C, 0.5C, C, where C is the maximal specified load). The signal swing is taken as the difference between minimum and maximum when the rolling elements move over the sensors. For the measured signal the mean and one standard deviation is indicated (sampling from two sensor pairs on two steel inlays with three repetitions). For both simulation and measurements the sensor response is largely linear w.r.t. to the external force and show excellent agreement in general.

The signal in movement is a measure for the spatial agreement of simulation and measurements, i.e., distribution of strains under the rolling element, showing excellent matching. In Figure 10 it should be mentioned that the distance of rolling elements in the FEM simulation was 5.3 mm (i.e., 0 mm more than the minimum distance). Additional noise is introduced in the measurement by a slightly varying distance between the rolling elements.

#### 2.2.6. Estimation of Roller Locations

**Figure 12:**
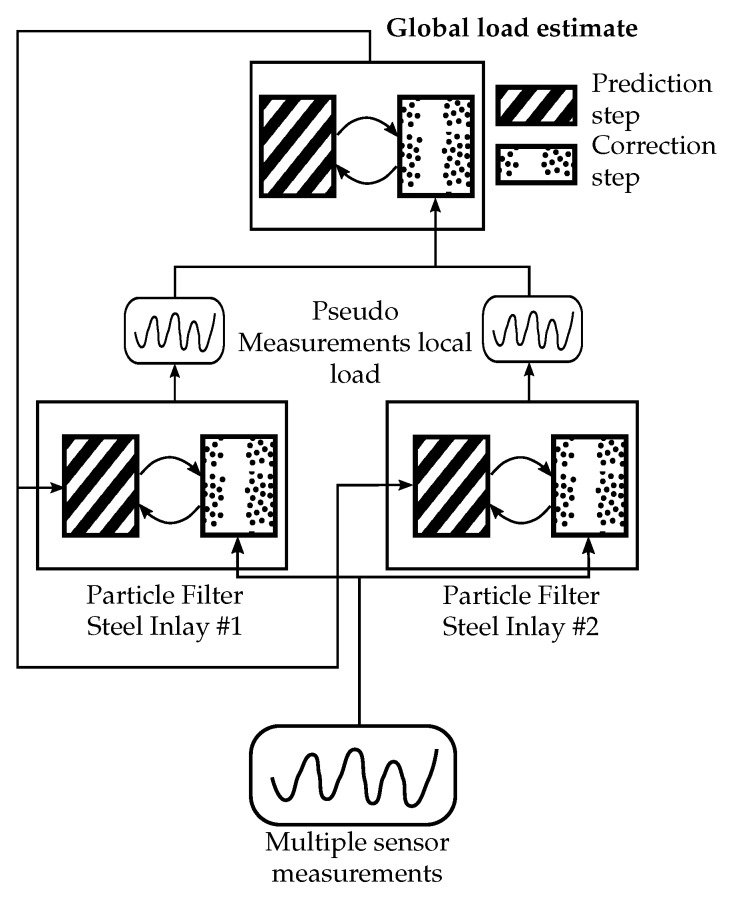
Block diagram of the employed estimator system for basic sensor fusion and load estimation.

While measuring the strain directly introduced by the rolling elements can be advantageous by being close to the point of load transfer, it also provides the challenge of signal dependency on the relative rolling element position, although this can also be exploited for signal correction, as shown above. In order to demonstrate real practical applicability of the load measurement principle, an algorithm estimating both the roller locations and the force acting upon the carriage was developed. The challenge is to estimate the force under roller location uncertainty, for two rollers, from only two difference signals per steel inlay. This clearly leads to an under determined and nonlinear equation system. For the estimation per steel inlay, in order to cope with the nonlinearities, a particle filter was employed. This filter produces a statistical estimate in the three dimensional state space. These two estimates, one per steel inlay in the force flux, are then taken as pseudo-measurements for a superimposed Kalman filter. The Kalman filter estimate is then fed back to the state space exploration, or prediction, part of the local particle filters. This way, sensor fusion over the four difference signals is realized. The exact implementation would be beyond the scope of this paper, the working principle is shown in Figure 12. For the mapping of the measurements to the state space, either a phenomenological function or one based on Section 2.2.1 can be used.

**Figure 13:**
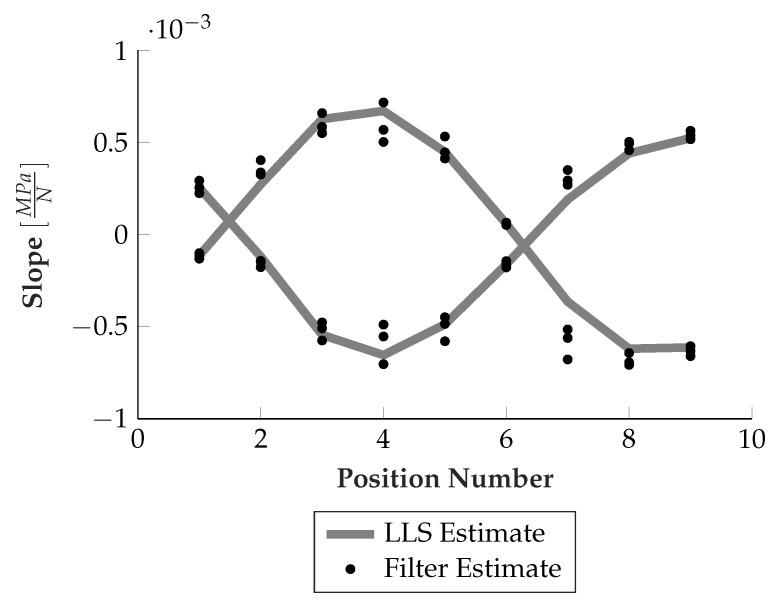
Slope estimation performance.

**Figure 14:**
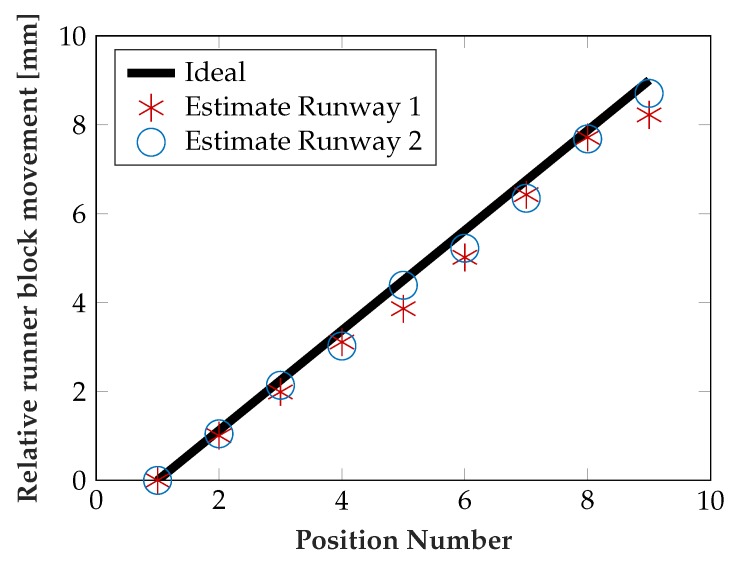
Position estimation performance.

To test the performance of the algorithm, the algorithm was provided with sensor data from nine different runner block positions, 1 mm apart each, again with increasing load from 0–100 kN. The measurements were repeated three times. Since the behavior for a given position is nearly linear, the estimated slopes were compared with a linear least squares (LLS) fit (i.e., virtually known position), the result is depicted in Figure 13. Also the relative runner block movement can be extracted from the estimated position of one of the rolling elements, which is shown in Figure 14.

Both estimates delivered fairly accurate results, with only some outliers, e.g., at position 7. Taking into account the underlying difficult estimation task as described above, this indicates real applicability of the technology even for more complex and dynamic load situations, if the sensor layout and algorithm is further developed.

#### 2.2.7. Remaining Non-Ideality: Hysteresis

**Figure 15:**
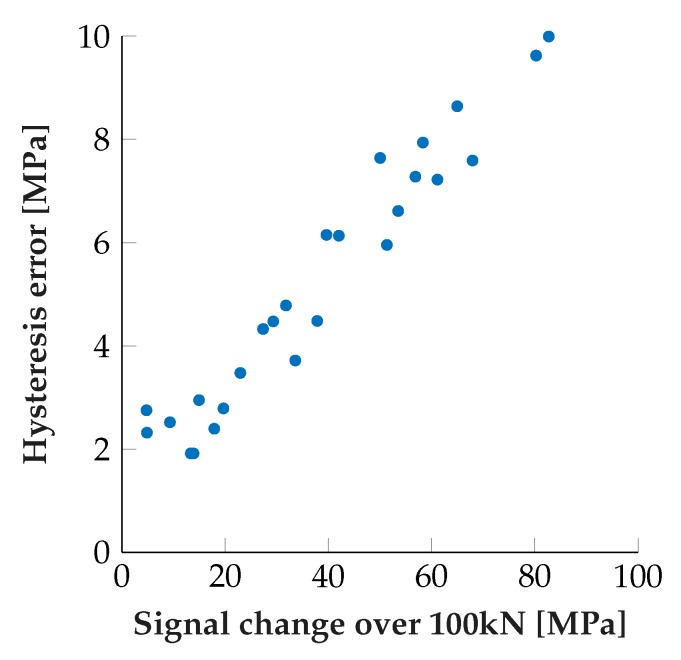
Correlation of hysteresis error to signal swing of two sensor pairs at 13 different roller positions.

The remaining non-ideality is hysteretic, in a sense that the sensor signals differ significantly when increasing or decreasing the external load on the runner block. The hysteresis magnitude, i.e., the maximum difference between sensor signal under increasing and decreasing load was found to be highly correlated to the roller dependent signal swing (Pearson correlation coefficient of approx. 0.97), as visible in Figure 15. This relationship raises the prospect of being able to predict both quantities from one estimator in later applications, where the position of the rolling elements is unknown at first.

A hysteresis of this magnitude definitely has to be compensated by either, if possible, modifications to the sensor layout or software based approaches. For both, research into the exact nature and ideally origin of the hysteresis is needed.

## 3. Discussion

The need for a reliable, standardized tooling machine component which actively measures the load inside the machine was shown by capturing the current state of technology and science. Methods for load measurement in linear guides were shown. A new approach using sensors for measuring the stress introduced by the rolling element contacts inside the linear guide bearing was deemed to be most fitting to the goal of integrating a load measurement system into a standard machine component, without interfering with the mechanical properties. The proposed system was evaluated theoretically and empirically. Theoretical evaluation was based on an analytical solution of a simplified problem. The results agreed well with FEM simulation data. This analytical model can now be used to predict and optimize sensor performance, also in related problems. Further, it can form the basis for an estimator to actually calculate the load out of the sensor signals. Critical cross-sensitivities were found, but also an easy to use compensation method using sensor signal differences was introduced and shown to be well performing. In order to exploit the conceptual advantages of measuring the stresses close to the rolling elements, it has to be shown that the sensors actually and only measure this stress. This was shown by comparison with FEM simulation data. The compensated signal showed good agreement with FEM simulations, and thus also with the analytical model. To show practical applicability of the sensor system, a method for estimating the roller location was proposed and successfully tested within a statistical estimator structure.

Following these promising results with a simple difference signal, it can be concluded that the measurement method and location of sensors, together with the proposed evaluation principle, are suitable for load measurement on a runner block, by both theoretical and practical aspects. Showing this was the purpose of this paper.

More complex sensor layouts for even better compensation and higher sensitivity will be investigated. The remaining dominant non-ideality is hysteretic. Further research into possible origins, modeling and compensation methods for the hysteresis is necessary for the successful deployment of the sensor technology.

## Abbreviations

The following abbreviations are used in this manuscript:

FEM	Finite element method
LDO	Low dropout (voltage) regulator
